# Targeting Inflammatory Signaling in Prostate Cancer Castration Resistance

**DOI:** 10.3390/jcm10215000

**Published:** 2021-10-27

**Authors:** Shangwei Zhong, Changhao Huang, Zhikang Chen, Zihua Chen, Jun-Li Luo

**Affiliations:** 1 Department of General Surgery, Xiangya Hospital, Central South University, Changsha 410008, China; swzhong@csu.edu.cn (S.Z.); Chhuang@csu.edu.cn (C.H.); 403445@csu.edu.cn (Z.C.); 2 Department of Molecular Medicine, The Scripps Research Institute, Jupiter, FL 33459, USA

**Keywords:** androgen deprivation therapy (ADT), castration-resistant prostate cancer (CRPC), inflammatory signaling, nuclear factor-kappa B (NF-κB), IκB kinase (IKK), macrophage, B-cells, T-cells, inflammatory cells, constitutive NF-κB activation, IKK/NF-κB inhibitors, androgen-dependent (AD), androgen-independent (AI)

## Abstract

Although castration-resistant prostate cancer (CRPC) as a whole, by its name, refers to the tumors that relapse and/or regrow independently of androgen after androgen deprivation therapy (ADT), untreated tumor, even in early-stage primary prostate cancer (PCa), contains androgen-independent (AI) PCa cells. The transformation of androgen-dependent (AD) PCa to AI PCa under ADT is a forced evolutionary process, in which the small group of AI PCa cells that exist in primary tumors has the unique opportunity to proliferate and expand selectively and dominantly, while some AD PCa cells that have escaped from ADT-induced death acquire the capability to survive in an androgen-depleted environment. The adaptation and reprogramming of both PCa cells and the tumor microenvironment (TME) under ADT make PCa much stronger than primary tumors so that, currently, there are no effective therapeutic methods available for the treatment of CRPC. Many mechanisms have been found to be related to the emergence and maintenance of PCa castration resistance; in this review, we focus on the role of inflammatory signaling in both PCa cells and the TME for the emergence and maintenance of CRPC and summarize the recent advances of therapeutic strategies that target inflammatory signaling for the treatment of CRPC.

## 1. Introduction

Prostate cancer (PCa) is the most common malignancy and the second-leading cause of cancer-related mortality in men in Western countries [1,2]. In tumors confined to the prostate, radical prostatectomy and radiotherapy are effective; however, for late-stage disseminated disease, current therapies are merely palliative [1,2]. Androgen receptor (AR) signaling is a critical survival pathway for PCa cells, and androgen deprivation therapy (ADT) is an initial systemic therapy for advanced PCa. It is also used as an adjuvant to local therapy for high-risk diseases. Although a majority of patients initially respond to ADT, the responses in advanced disease are transient, and almost all eventually develop castration resistance [3,4]. Castration-resistant PCa (CRPC) is associated with a very poor prognosis, the treatment of which remains a serious clinical challenge [1,2,5,6]. Understanding the mechanisms that underlie the pathogenesis of castration resistance is, therefore, needed to develop novel therapeutic approaches for this disease.

Many mechanisms for castration resistance have been proposed [5,7,8,9,10]: the continuous role of androgen receptor (AR) signaling in CRPC due to the amplification/mutation of ARs or the increased expression of AR splice variants or androgen synthesis enzymes in PCa cells [5,6,11]; the activation of other signaling transduction pathways that lead to either the enhanced activity of ARs and its coactivators or the bypassing of ARs in the presence of low levels or even in the absence of androgen [5,6,12]; and the existence of PCa stem cells that do not depend on ARs to survive [5,6]. We and many others have reported that immune/inflammatory signaling in both cancer cells and the tumor microenvironment (TME) plays important roles in CRPC growth and development [13,14,15,16,17].

Inflammatory signaling activation has been found in almost all cancers and is associated with cell transformation, tumorigenesis, tumor progression, and therapy resistance [14,15,16]. NF-κB transcription factors play crucial roles in the regulation of inflammation, immune responses, and survival of both normal and malignant cells. There are two traditional NF-κB activation pathways. The activation of the classical NF-κB pathway is triggered by the activation of the tripartite IKK complex, composed of IKKα, IKKβ, and IKKγ/NEMO, while the alternative NF-κB pathway is mediated by the selective activation of IKKα [18,19]. Accumulated evidence suggests that inflammatory signaling, both in PCa cells and in the TME, involves the initiation and maintenance of CRPC and is associated with adverse outcomes of PCa patients [7,20,21]. For instance, IKKβ or IKK complex-independent constitutive NF-κB activation promotes castration-resistant PCa development [22,23]. IKKα activation by RANK ligand promotes PCa metastatic progression through the inhibition of Maspin expression in PCa cells [24]. In addition, the inflammatory signaling in the TME also contributes to CRPC development [13,25]. Given the crucial roles of inflammatory signaling in CRPC development, more and more targeted therapeutic strategies have emerged to interrupt inflammatory signaling in PCa cells and in the TME. Here, we briefly review the recent advances in the roles of inflammatory signaling in the emergence and maintenance of CRPC as well as the therapeutic strategies that target inflammatory signaling for the treatment of PCa castration resistance.

## 2. Inflammatory Signaling in PCa Cells in PCa Castration Resistance

**Figure 1:**
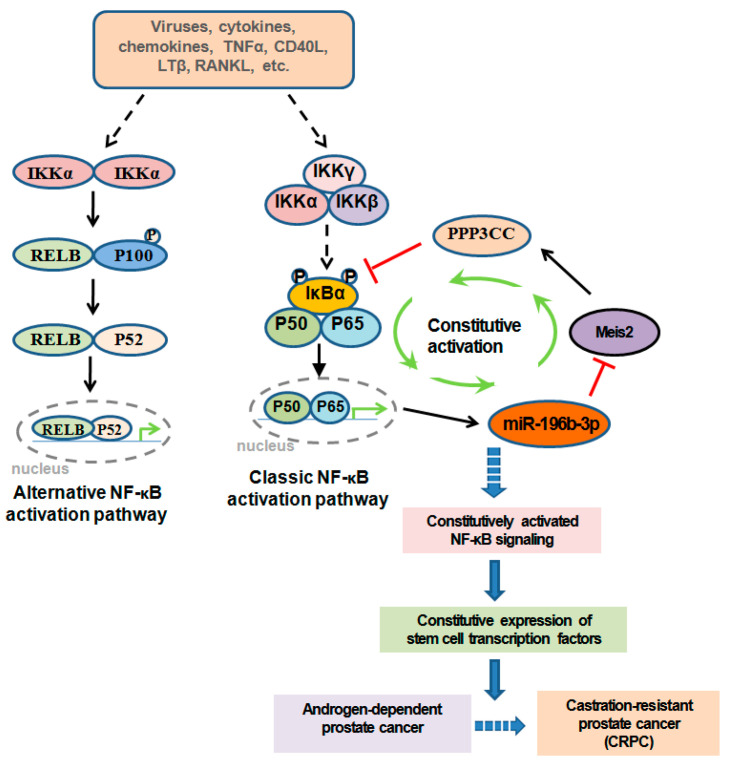
Diagram of traditional NF-κB pathway and the constitutively activated feed-forward signaling circuit in CRPC. A constitutively activated feed-forward circuit, composed of IkBα/NF-κB(p65), miR-196b-3p, Meis2, and PPP3CC, formed during and required for CRPC development. Constitutive activation of this circuit is independent of traditional IKKβ/NF-κB pathways, suggesting that targeting this circuitcould be important for CRPC treatment.

For PCa, AR signaling is a crucial survival pathway; however, PCa cells are forced to search for other lifelines to survive in an androgen-depleted environment under ADT. Accumulating evidence suggests that inflammatory signaling in both PCa cells and the TME is one of these important lifelines that promote the emergence and maintenance of CRPC. After androgen ablation, cytokines and growth factors such as RANKL activate IKKα and STAT3 in PCa cells that produce chemokines, such as CXCL13, to attract the infiltration of leukocytes, which facilitate PCa cell androgen-independent growth and survival [13]. It has been reported that the classical NF-κB signaling pathway is constitutively activated in CRPC cells [26,27]. We recently showed that CRPC cells express much higher levels of stem cell markers and have much stronger tumorigenicity than androgen-sensitive PCa. Our study further demonstrated that IKKβ or IKK complex-independent constitutive NF-κB activation supports the survival of PCa cells after androgen ablation, leading to CRPC development [22,23]. Activation of this intrinsic constitutively activated NF-κB signaling circuit, composed of miR-196b, Meis2, PPP3CC, and p65, drives tumorigenicity and CRPC development in allograft/xenograft mouse models and is manifested in human prostate tumors (Figure 1). Complementary loss-of-function and gain-of-function experiments have shown that targeting the non-IκBα/NF-κB (p65) individual components of this feed-forward loop interrupts this constitutive activation of NF-κB and significantly impairs CRPC development. Very importantly, constitutive p65 activation in the signaling circuit is independent of traditional NF-κB pathways, which set a specific window for the treatment of CRPC; this will avoid the severe side effects related to general IKKβ/NF-κB inhibition in normal cells [22]. The constitutive NF-κB signaling circuit drives the expression of a group of stem cell transcription factors, including Twist2, Sox2, Oct4, and Nanog, to promote the cancer stem cell-like characteristics and tumorigenicity of CRPC cells [22]. Consistently, a recent report supported the role of MEIS2 in cancer cell stemness [28].

**Figure 2:**
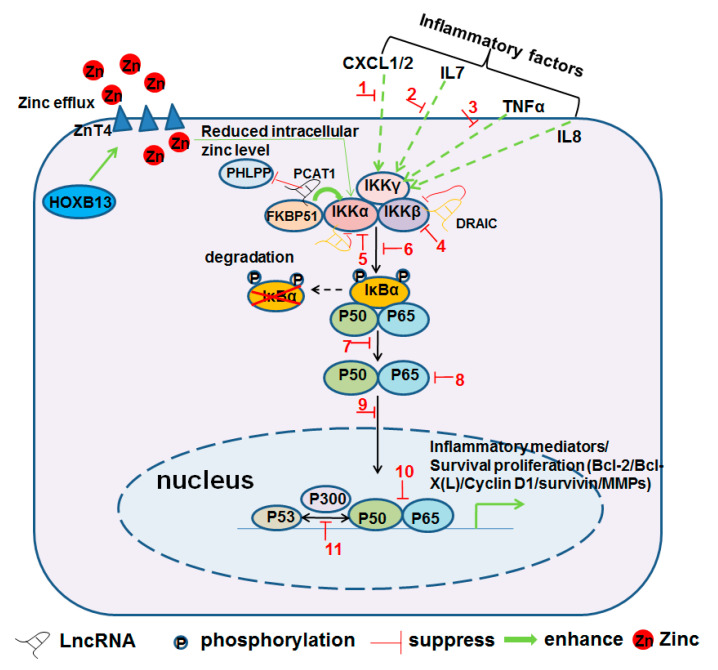
NF-κB inflammatory signaling as a contributor to CRPC development and the therapeutic strategies for the suppression of CRPC by targeting NF-κB inflammatory signaling. Note: 1: curcumin; 2: thymoquinone; 3: ursolic acid/apigenin/α-tomatine; 4: CmpdA/EC-70124/curcumin; 5: apigenin; 6: imipramine/ursolic acid/apigenin/retigeric acid B/aspirin/α-tomatine/simvastatin/ acacetin/betulinic acid; 7: artesunate; 8: polyphyllin I; 9: retigeric acid B/aspirin/pao pereira extract/α-tomatine/simvastatin/betulinic acid/curcumin; 10: ursolic acid/diosgenin/pao pereira extract/DIM/betulinic acid; 11: NQO1.

In addition, it has been reported that high mobility group box 1 protein (HMGB1) activates the NF-κB signaling pathway via interaction with and binding to TNFR1 in CRPC cells [29]. NAD(P)H:quinone oxidoreductase 1 (NQO1) links redox homeostasis with inflammatory signaling in PCa cells. NQO1 inhibition can reinforce survival signaling under conditions of androgen deprivation by promoting interactions between NF-κB and p300, which increase the accumulation of nuclear IKKα and NF-κB while decreasing the expression of p53 protein [30]. HOXB13 is overexpressed in CRPC cells and promotes CRPC cell invasion and metastasis. The overexpressed HOXB13 upregulates Znt4, a zinc efflux channel, to reduce intracellular zinc concentrations, which then stimulates IKKα expression and IκBα degradation to enhance the nuclear translocation of RelA/p65 in PCa cells (Figure 2) [31,32].

Long noncoding RNAs (LncRNA) have been reported to be associated with PCa progression [33,34]. Among them, some LncRNAs act as specific regulators of inflammatory signaling in CRPC and may be potentially novel targets for CRPC treatment. It has been reported that LncRNA PCAT1 contributes to the activation of AKT as well as NF-κB signaling through the perturbance of the PHLPP/FKBP51/IKKα complex in CRPC; targeting LncRNA PCAT1 significantly suppresses CRPC development [35]. Another LncRNA DRAIC, which is down-regulated in CRPC, acts as an inhibitor of NF-κB. DRAIC expression and is associated with poor outcomes in PCa patients. DRAIC interacts with the IKK complex and inhibits its activation, which inhibits IκBα phosphorylation and NF-κB activation (Figure 2) [34].

Pro-inflammation cytokines and chemokines in the inflammatory TME support the CRPC initiation and progression, while CRPC cells are able to produce their own cytokines, chemokines, and growth factors [20,36]. For instance, pro-inflammatory chemokine interleukin-8 (IL8) from both PCa cells and the TME promotes CRPC initiation, progression, and metastasis. IL8 treatment decreases the sensitivity of LNCaP and LAPC-4 cells to anti-androgen agents and reduces their dependence on androgen for growth. IL8 treatment reduces PSA and AR levels while upregulating AKT and NF-κB activity [37]. Inflammatory mediator IL6 is associated with STAT3 activation in PCa cells and is implicated in the transition of androgen-dependent PCa to CRPC. Both IL8 and IL6 enhance the infiltration of MDSCs into the TME, which facilitates the constitution of a highly immunosuppressive microenvironment of CRPC tumors [38]. It is reported that an autocrine/paracrine feedback loop, linking pro-inflammatory cytokines CXCL1 and -2 to NF-κB in PCa cells, regulates PCa cell growth and metastasis. Treatment with curcumin abolishes this pro-inflammatory and pro-metastatic feedback loop and reduces lung metastasis [39]. It has been reported that elevated TNFα is associated with PCa castration resistance and the poor overall survival of PCa patients [40]. Treatment with TNFα increases phospho-p65 levels and activates NF-κB signaling in PCa cells (Figure 2) [41].

## 3. Inflammatory Signaling in the TME in PCa Castration Resistance

The inflammatory microenvironment of neoplastic tissue can be identified by the presence of host leukocytes, which consists of macrophages, dendritic cells, mast cells, B-cells, and T-cells [42]. Tumor-associated immune/inflammatory cells are crucial cellular components in the TME. The cross-talk between tumor-associated immune/inflammatory cells and PCa cells drives PCa progression, metastasis, and castration resistance.

### 3.1. Inflammatory Signaling and Myeloid Cells

Tumor-associated immune/inflammatory cells, including myeloid-derived suppressor cells (MDSCs) and tumor-associated macrophages (TAMs), not only constitute an immunosuppressive TME that helps and accommodates cancer cells escaping from immune surveillance but also produces pro-inflammatory cytokines, chemokines, and growth factors to promote tumor growth and progression and to counter any stress and insult conditions, such as chemo/radiotherapy and ADT. Meanwhile, inflammatory factors, such as CCL2 and CXCL2, facilitate the recruitment and infiltration of tumor-associated immune/inflammatory cells, such as TAMs and MDSCs, into the TME [20,43,44].

TAMs, classified into M1 and M2 types, are frequently found within the TME; they are important sources of cytokines, which promote tumor growth, progression, and therapy resistance. M1 macrophages are classically activated by interferon-γ (IFN-γ) and microbial products, killing microorganisms and expressing high levels of pro-inflammatory cytokines (TNFα, IL-1, IL-6, and IL-12) and reactive oxygen and nitrogen intermediates. In contrast, M2 macrophages tune inflammation and adaptive immunity by down-regulating MHC class II and IL-12 expression and upregulating IL-10, scavenger receptor A, and arginase [43,45,46]. Most TAMs are associated with M2 macrophages in promoting tumor angiogenesis and tissue remodeling [47,48,49]. TAM infiltration is correlated with PCa Gleason scores and serum PSA levels in PCa patients and is associated with increased recurrence rates and poor prognosis [50,51,52,53].

Inflammatory signaling is essential for the maintenance of the pro-tumoral activities of TAMs and MDSCs. It has been reported that NF-κB plays a major role in maintaining the immunosuppressive phenotype of TAMs. The polarization of macrophages to an immunosuppressive “alternative” phenotype via interleukin (IL)-1R and MyD88 requires IKK-mediated NF-κB activation [54]. A novel Mincle/Syk/NF-κB signaling pathway has been recently proposed in M2-like TAM; disruption of this signaling pathway suppresses the expression of NF-κB-regulating genes, such as the pro-tumoral inflammatory cytokine IL6 [55]. It has also been reported that the immunosuppressive activities of MDSCs, mediated by transmembrane TNFα (tmTNFα), are associated with the activation of NF-κB. Inhibition of NF-κB abrogates the tmTNFα-mediated suppression of lymphocyte proliferation [56]. CXCL2 secreted from PCa cells promotes the infiltration of TAMs to the TME and polarizes macrophages to an anti-inflammatory phenotype through C-X-C chemokine receptor type 2 (CXCR2). Blockade of CXCR2 receptors in TAMs with AZD5069 disrupts the CXCL2-CXCR2 pathway and triggers the reprogramming of TAMs toward a pro-inflammatory state, resulting in PCa cell senescence and the inhibition of tumor progression [53]. In hormone-refractory PCa PC3 cells and xenograft mouse models, IL4 stimulates TAMs to secrete pro-angiogenic and pro-tumor chemokine expression, such as CCL2, to promote angiogenesis and PCa growth [57].

### 3.2. Inflammatory Signaling and Infiltrated Lymphocytes

The infiltration of lymphocytes is increased in late-stage and recurrent PCa, and the infiltration of B and T lymphocytes is very common in almost all human PCa samples with high Gleason scores [58,59,60,61]. A pre-clinical model demonstrates that androgen ablation elicits a tumor-associated inflammatory response. IKKβ and NF-κB activation in the infiltrated B-cells by mediators released from necrotic cell death upregulates the expression of inflammatory cytokine lymphotoxin (LT), which, in turn, stimulates IKKα and STAT3 activation in PCa cells to enhance androgen-independent (AI) growth and survival [13,62]. Plasmocytes (IgA^+^, expressing-IL-10 and PD-L1) is a critical immunosuppressive subset of B-cells that blocks tumor-directed CD8^+^ cytotoxic T-cell activation in CRPC. Elimination of IgA^+^ plasmocytes promotes the response of CRPC to oxaliplatin [63].

Both stromal cells and immature B-cells in bone marrow express ARs, and immature B-cells are positive for ligand-binding and cytoplasmic AR protein in contrast to mature B-cells in the spleen, which do not express receptors for androgens [64,65]. Androgen deprivation by castration in normal mice leads to the expansion of B-cell populations in the spleen and bone marrow, whereas androgen replacement in castrated mice reverses the effects only in bone marrow but not in the spleen in the case of negative AR expression. Moreover, AR mutations in mice also increase B-cells in the peripheral blood [66,67,68]. These indicate that androgens and AR expression have a decisive effect on B-cell development within bone marrow but not within the spleen. After castration, immune cells such as T- and B-cells and NK cells infiltrate into the regressing prostate tumors, and, notably, lymphotoxin (LT) derived from B-cells, but not from T-cells, control the recurrence of CRPC, which is mediated by LTβR and induces IKKα nuclear translocation and STAT3 activation [13]. The activated STAT3 facilitates the translocation of ARs to the nucleus in the absence of androgens [69]. These findings suggest that the expansion of B-cell populations by castration is involved in promoting CRPC development; however, in localized PCa, increased plasma cells are associated with improved outcomes [61,63,70,71,72].

T-cells are generated in the thymus and by peripheral expansion. At the early stage, T-cell progenitors migrate from bone marrow to the thymus, and they mature through several sequential stages. Mature T-cells can be divided into two groups based on the expression pattern of T-cell receptors (TCRs) which are γδ and αβ. αβT-cells are subdivided into CD8^+^ cytotoxic T-cells (CTLs) and CD4^+^ helper T (Th) cells according to their effector functions, and Th cells include Th1, Th2, Th17, T regulatory (Treg) cells, and natural killer T (NKT) cells [73,74]. The infiltration of T-cells, which are predominantly composed of Th cells and comparatively fewer CTLs, increases in response to immunization against prostate tumor antigens. Th cells promote an anti-tumor response, and Th cells play an important role in engaging with antigen-presenting cells (APCs) and, thus, activating the CTLs. The augmentation of CD8^+^ T-cells plays an important role in the homing of antigen-specific CTLs, and they induce the production of interleukin-2 (IL-2) by the activated cells, which increases precursors by engaging the IL-2 receptors on responder cells [75]. Androgen levels directly affect thymic epithelial and indirectly the development of T-cells in the thymus, which are related to AR expression in thymic epithelium. Consistently, ADT leads to an increase in thymopoiesis and reverses thymic atrophy in post-pubertal male mice; the administration of testosterone decreases thymopoiesis. ADT also induces T-cell infiltration to the area of prostate tumors and T-cell priming to prostatic antigens, which increase several APCs, such as macrophages and dendritic cells, in the tissue levels. ADT increases both CD8^+^ T-cells and Tregs in the TME; the degree of infiltration of CCR4+ Tregs is related to the prognosis of PCa patients, suggesting that a combination of Treg-depleting agents may improve the efficacy of ADT for the treatment of advanced PCa [58,60].

### 3.3. Inflammatory Signaling and the ECM Network of the TME

The TME is composed of cellular and non-cellular components. The extracellular matrix (ECM) is a major non-cellular component of the TME that is constructed by more than one hundred proteins that are organized into a structural framework, which acts as a scaffold for (tumor) cells [43]. Together with other components in the TME, PCa cells are able to interact with the ECM to establish a chronic inflammatory and immunosuppressive environment to support CRPC growth and development. Extracellular matrix factors promote PCa cell androgen-independent (AI) growth and CRPC development [76,77]. A remodel of the ECM, mediated by matrix metalloproteinases (MMPs), is closely associated with CRPC metastasis and therapeutic resistance [78]. MMPs, a major mediator of the ECM, can enhance the degradation of components of the ECM, such as collagen [79,80]. MMP activity can be regulated by inflammatory signaling in PCa cells. It has been reported that matrix metalloproteinase-9 (MMP-9) activity is associated with NF-κB activation in PCa cells. Aspirin treatment causes a strong decrease in IKKβ activation, IκBα phosphorylation, and p65 nuclear translocation, leading to reduced MMP-9 activity and urokinase-type plasminogen activator (uPA) and plasminogen activator inhibitor-1 (PAI-1) expression. Consequently, cell invasion and attachment of DU145 and PC3 cells are suppressed. Moreover, IKKβ overexpression can reverse the inhibitory effects of aspirin on PCa cell invasion [81]. MMP-3 and MMP-7 drive the IL-7-induced tumor progression, migration, and invasion of DU-145 cells. Thymoquinone (Tq) treatment suppresses the activation of MMPs via IL-7/AKT/NF-κB signaling that inhibits IL-7-induced tumor progression and metastatic invasion in DU145 cells [82].

## 4. Therapeutic Strategies for Targeting Inflammatory Signaling in CRPC

Recently, many inhibitors that target inflammatory signaling for the treatment of CRPC have emerged; some of them have been in clinical trials [7]. Here, we summarize some recent therapeutic strategies designed to suppress CRPC development by targeting NF-κB inflammatory signaling in CRPC cells, immune cells, and the ECM (Figure 2 and Table 1).

### 4.1. Imipramine

Imipramine(10,11-dihydro-*N*,*N*-dimethyl-5H-dibenz[b,f]azepine-5-propanaminehydrochloride), a member of the TCA family, attenuates PCa proliferation, migration, and invasion in PC-3 cells and xenograft mouse models. Imipramine inhibits IκBα phosphorylation and p65/RelA nuclear translocation, resulting in reduced secretion of some pro-inflammatory chemokines and cytokines, including TNFα and IL1β [83].

### 4.2. Artesunate

Artesunate (AS) and other artemisinin derivatives have been identified as anti-cancer agents due to its anti-proliferative, anti-angiogenic, and anti-inflammatory properties [101]. AS can target NF-κB signaling; when combined with bicalutamide (Bic), AS, and Bic, it attenuates the oncogenic properties of CRPC cells and sensitizes CRPC cells to androgen receptor (AR) antagonists through the inhibition of NF-κB signaling and decreases AR and/or AR-variant 7 expression. A PC3 xenograft model shows that the combination of AS and Bic results in remarkable tumor regression and reduces lung and bone metastases [41]. AS has already been applied in clinical trials for the treatment of colorectal cancer (ISCRTN05203252), metastatic breast cancer (NCT00764036, phase I), and advanced lung and gastrointestinal cancer (NCT02353026, phase I) [102,103,104]. In the completed clinical trials for colorectal cancer, AS showed anti-proliferative activity in cancer cells and is generally well tolerated for the patients [102]. In the trial of NCT02353026, intravenous administration of AS was performed; the maximum tolerated dose (MTD) is 18 mg/kg, and the treatment was well tolerated [104]. These clinical trials’ data may provide some clues for the application of AS for the treatment of CRPC.

### 4.3. Pao Pereira Extract

Pao pereira extract is derived from bark of Amazonian tree Pao Pereira. Pao pereira extract treatment can suppress PC3 cell growth by the induction of cell cycle arrest and apoptosis. Pao pereira extract also blocks PC3 cell migration and invasion. Pao pereira extract suppresses NF-κB signaling by inhibiting NF-κB/p65 nuclear translocation and its transcription activity in the cells. The growth inhibitory effect of Pao extract can be reversed by the overexpression of RelA/p65 [84].

### 4.4. Polyphyllin I (PPI)

Polyphyllin I (PPI) is a steroidal saponin in Paris polyphylla and exhibits anti-tumor effects in several cancers, including PCa. PPI can significantly inhibit CRPC growth by the induction of cell cycle arrest, which is mediated by the down-regulation of p65 and MUC1 protein expression as well as LncRNA HOX transcript antisense RNA (HOTAIR). The combination of PPI and enzalutamide shows a synergistic inhibitory effect in CRPC (PC3 and DU145) in vitro and in vivo [85].

### 4.5. CmpdA

CmpdA, an IKKβ inhibitor, inhibits constitutively activated IKKβ/NF-κB signaling and IKKβ/Nanog signaling in CRPC. As a result, CmpdA treatment leads to the inhibition of proliferation, migration, and stemness and the induction of apoptosis in PC3 and DU145 cells and xenograft mouse tumors. The combination of CmpdA with docetaxel exhibits a synergistic inhibitory effect in PC3 and DU145 cells [86].

### 4.6. EC-70124

EC-70124, a novel glycosylated indolocarbazole compound, is a multikinase inhibitor with potent activity against IKKβ and JAK2 [87,105,106]. EC-70124 exhibits a concomitant inhibition of NF-κB and STAT3 in CRPC cells. EC-70124 treatment significantly decreases cell proliferation, migration, and colony formation of DUI45 in vitro and in xenograft mouse models. The underlying mechanism is that EC-70124 blocks the concomitant activation of NF-κB and STAT3 by reducing the phosphorylation of IkBα and STAT3 (Tyr705) as well as inhibiting p65 nuclear translocation [87].

### 4.7. Ursolic Acid

Ursolic acid (3β-hydroxy-urs-12-en-28-oic-acid), a pentacyclic triterpenoid, belongs to the cyclosqualenenoid family. Ursolic acid has been reported to exhibit an inhibitory effect on several cancer cells [107,108]. Ursolic acid suppresses DU145 cell proliferation in a dose- and time-dependent manner in vitro and tumor development of DU145 xenograft mouse models. Ursolic acid inhibits TNFα-induced and constitutive IKK activation in DU145 cells. In addition, ursolic acid inhibits constitutive STAT3 phosphorylation and Src and JAK2 kinase activation [88].

### 4.8. Apigenin

Apigenin, a common plant flavonoid, can reverse the resistance of androgen-insensitive prostate cells to TNFα-induced apoptosis through the inhibition of NF-κB signaling. Apigenin significantly decreases IKK activity, IκBα phosphorylation and degradation, and nuclear NF-κB, leading to the down-regulation of many NF-κB-regulating genes, such as Bcl2, COX-2, MMP-9, NOS-2, and VEGF, which sensitize hormone-refractory cancer cells to TNFα-induced apoptosis [89].

### 4.9. Retigeric Acid B

Retigeric acid B is a natural pentacyclic triterpenic acid derived from lichen. Retigeric acid B exhibits an inhibitory effect on cell growth and induces apoptosis in androgen-independent PCa cells. Retigeric acid B-mediated inhibitory effects on PC3 and DU145 cell proliferation and tumor growth are attributed to its suppression of NF-κB signaling. Retigeric acid B inhibits the phosphorylation of IκBα and p65 and blocks p65 nuclear translocation and DNA binding activity, which lead to the down-regulation of NF-κB-regulating genes, including Bcl-2, Bcl-x(L), cyclin D1, and survivin [90].

### 4.10. α-Tomatine

Alpha (α)-tomatine, a major saponin present in tomato, inactivates NF-κB signaling to inhibit hormone-refractory PCa cell proliferation in vitro and xenograft tumor development in mouse models. The α-tomatine inhibits IKK activity that leads to the suppression of IκBα phosphorylation and degradation, NF-κB/p65 phosphorylation, and nuclear translocation, resulting in the decreased expression of NF-κB-dependent anti-apoptotic proteins such as c-IAP1, c-IAP2, Bcl-2, Bcl-xL, XIAP, and survivin. In addition, α-tomatine treatment reduces TNF-α-induced activation of the pro-survival mediator AKT [91,109].

### 4.11. Simvastatin

Simvastatin, a 3-hydroxy-3-methylglutaryl coenzyme A (HMGCoA) reductase inhibitor, exhibits inhibitory effects on the cell growth of hormone-refractory PCa PC3 and DU-145 cells by inhibiting the NF-κB pathway. Simvastatin treatment inhibits IκBα phosphorylation and degradation and reduces phosphorylated p65 protein levels in nuclei, leading to the down-regulation of cIAP-1 and 2, cFLIP-S, and XIAP expression [93,110]. Simvastatin has been included in several clinical trials combined with other anti-cancer drugs, including XELOX, bevacizumab (NCT02026583), and capecitabine–cisplatin (NCT01099085); however, the addition of simvastatin, especially at the low dose (40 mg), does not increase the benefits [111,112]. A planned clinical trial of simvastatin, together with metformin for prostate cancer, was withdrawn due to low enrollment (NCT01561482) [7].

### 4.12. Acacetin

Acacetin (5,7-dihydroxy-4′-methoxyflavone), a flavonoid compound, has anti-inflammatory and anti-cancer effects. Acacetin can decrease cell viability and induce apoptosis in DU145 cells in vitro and suppresses DU145 xenograft growth in mouse models. Acacetin inhibits the phosphorylation of IκBα and NF-κB, leading to the reduced expression of NF-κB, which regulates anti-apoptotic proteins such as Bcl-2, XIAP, and COX-2 [94].

### 4.13. Metformin

Metformin acts as an anti-cancer agent in various types of cancers, including PCa. Metformin suppresses N-cadherin independently of AMPK that affects NF-κB activity in cells. Metformin-induced apoptosis is associated with the loss of p65 accumulation. The levels of N-cadherin and p65 in radical prostatectomy tissue can predict the effectiveness of metformin for the treatment of PCa post-surgery patients [95]. Metformin is a promising compound for prostate cancer therapy and is included in a group of clinical trials for prostate cancer [7]. One completed trial shows that metformin treatment yields disease stabilization and delays prostate-specific antigen doubling time in some patients with castration-resistant PCa (NCT01243385) [113].

### 4.14. 3,3′-Diindolylmethane (DIM)

3,3′-diindolylmethane (DIM), a major in vivo acid-catalyzed condensation product of indole-3-carbinol, exhibits protective effects against the development of (prostate) cancer. DIM synergizes Taxotere-induced apoptotic death in C4-2B CRPC cells. The combined DIM and Taxotere treatments inhibit C4-2B bone tumor growth. The enhancing effects are linked to decreased survivin expression and NF-κB DNA-binding activity [97]. A phase I clinical trial of oral DIM in castrate-resistant, non-metastatic prostate cancer showed modest efficacy and was well tolerated; a further phase II study was recommended with a dose of 225 mg orally twice daily [114].

### 4.15. Betulinic Acid

Betulinic acid (BetA), a pentacyclic triterpene from the bark of white birch, exhibits effective inhibition of NF-κB in hormone-refractory PCa cells. BetA significantly decreases the cell viability of PC3 in a dose- and time-dependent manner. BetA treatment decreases IKK activity and inhibits the phosphorylation of IκBα. BetA treatment also inhibits NF-κB/p65 nuclear translocation and DNA binding, leading to the reduced expression of NF-κB-regulating anti-apoptotic proteins. Consequently, BetA sensitizes the hormone-refractory PCa cells to TNFα-induced apoptosis [98].

### 4.16. Diosgenin

Diosgenin, a steroidal saponin derived from fenugreek, exhibits anti-cancer properties in various cancer cells. Diosgenin inhibits PCa cell proliferation, migration, and invasion. Diosgenin inhibits the activities and expression of matrix metalloproteinase-2 (MMP-2) and MMP-9 while increasing the expression of tissue inhibitor metalloproteinase-2 (TIMP-2). In addition, diosgenin suppresses extracellular signal-regulating kinase (ERK), c-Jun N-terminal kinase (JNK), and phosphatidylinositide-3 kinase (PI3K)/AKT signaling pathways as well as NF-κB activity [100].

## 5. Conclusions and Perspectives

The TME is rich in immune and inflammatory cells, including macrophages, T-cells, B-cells, dendritic cells (DC), and neutrophils. These cells interact with cancer cells directly or indirectly through a variety of mediators, including cytokines and chemokines. Based on our work and many others, it seems to be a chain reaction of events that leads to the development of AI PCa. This evolutionary process is initiated by the deaths of AD PCa following the depletion of androgen, which provokes the infiltration of immune and inflammatory cells. These cells then produce cytokines/chemokines that signal to androgen-insensitive PCa cells to promote their self-renewal, proliferation, and differentiation into AI cell types and the formation of hormone-refractory PCa. This evolutionary process is driven by cytokines and chemokines from immune/inflammatory cells in the TME, which are induced to differentiate into AI cell types. In turn, these AI cells acquire self-renewal properties, and they secrete some cytokines/chemokines, such as IL1, IL6, and IL8, and/or neuropeptides, such as neuroendocrine factors, to protect them from apoptotic insults.

Inflammatory signaling in both PCa cells and the TME plays crucial roles throughout every event of this reaction chain. NF-κB transcription factors, the key regulators of inflammatory signaling, play important roles in both the emergence and maintenance of CRPC. Currently, most NF-κB inhibitors are designed to target either NF-κB itself or the components of traditional NF-κB activation pathways (Figure 2). It should be noted that there are differences between NF-κB activation in acute immune responses and NF-κB constitutive activation in tumor cells, and there are differences between the activation of NF-κB pathways and the activation of NF-κB itself [22,23] (Figure 1). Our studies suggest that the constitutive activation of NF-κB in tumor cells is different from NF-κB acute activation in immune cells, and the constitutive activation of NF-κB in tumor cells does not depend on traditional NF-κB pathways, which are normally related to the activation of the IKKβ or IKK complex (Figure 1) [13,22]. This can explain why IKKβ/IKK inhibitors are not as powerful for the inhibition of tumor cell proliferation and tumor growth as was expected that they theoretically should do. Targeting the non-IκBα/NF-κB(p65) components in the constitutive NF-κB signaling circuit, including miR-196b-3p, Meis2, and PPP3CC, not only inhibits the constitutive NF-κB activation specifically in CRPC cells but also avoids the side effects by the indiscriminate inhibition of NF-κB in normal cells (Figure 1). Therefore, any strategies, particularly small-molecule inhibitors or activators, that target non-IκBα/NF-κB(p65) components in the constitutive NF-κB signaling circuit would be highly desirable.

Tumor-infiltrating lymphocytes (TILs) are key mediators of anti-tumor immune responses and maintenance. ADT increases CD8^+^ T-cells in the TME. However, some reports have shown that increased CD8^+^ T-cell infiltration correlates with a poor prognosis of PCa [115,116]. The complexity of the TME suggests that there would be important unknown mechanisms leading to the inhibition of T-cell killing and immune suppression in the TME of prostate cancer. Therefore, any strategies that target the TME to protect the anti-tumor immune function of CD8^+^ T-cells or to reverse the dysfunctional T-cells would improve the efficacy of ADT for the treatment of advanced PCa.

## Figures and Tables

**Table 1:** NF-κB pathway-related inhibitors that target inflammatory signaling in CRPC.

Target	Method or Inhibitor	Target Strategy	Mechanism of Action	Reference
NF-κB signaling pathway	Imipramine	attenuates PC-3 cell proliferation and inhibits migration and invasion in PC-3 cells	decrease p-IKK, p-IκBα, and p-p65	[83]
NF-κB signaling and AR and/or AR-variant 7 expression	Artesunate (combined with bicalutamide)	sensitize CRPC cells to antagonists, lead to tumor regression and reduce lungs and bone metastases	inhibition of NF-κB signaling and decrease AR and/or AR-variant 7 expression	[41]
NF-κB signaling pathway	Pao pereira extract	suppress CRPC PC3 cell growth, migration, and invasion	inhibit relocation of NF-κB/p65 in cells and NF-κB/p65 transcription activity	[84]
NF-κB/p65	Polyphyllin I (PPI) (combined with enzalutamide)	suppress CRPC cell growth and tumor development	decrease p65 and MUC1 protein expression as well as lncRNA HOX transcript antisense RNA (HOTAIR) expression	[85]
IKKβ	CmpdA (synergize with docetaxel)	inhibit cell proliferation, migration, and stemness and induction of apoptosis; suppress tumor growth	inhibit constitutively activated IKKβ/NF-κB signaling and IKKβ/Nanog signaling	[86]
NF-κB and STAT3	EC-70124	decrease cell proliferation, migration, and colony formation of DUI45; suppress tumor growth	block concomitant activation of NF-κB and STAT3	[87]
IκB kinase (IKK)	Ursolic acid	decrease DUI45 cell proliferation, suppress tumor growth	inhibit TNFα-induced and constitutive IKK activation as well as NF-κB-dependent reporter activity	[88]
IKKα kinase	Apigenin	sensitize PC3 cells to TNFα-induced apoptosis	decrease IKKα kinase activity and inhibit IκBα degradation and IκBα phosphorylation	[89]
IκBα and p65	Retigeric acid B	inhibit cell proliferation and tumor growth in PC3 and DU145 cells models	inhibit phosphorylation levels of IκBα and p65, and block the translocation of p65 to the nucleus and its DNA binding activity	[90]
IκBα kinase	α-tomatine	inhibit cell proliferation and attenuate the growth of subcutaneous and orthotopic xenograft tumors of PC3	inhibit IKK kinase activity, resulting in sequential suppression of IκBα phosphorylation, IκBα degradation, NF-κB/p65 phosphorylation, and NF-κB p50/p65 nuclear translocation; reduce TNFα-induced activation of the pro-survival mediator Akt	[91,92]
IκBα	Simvastatin	inhibit cell growth and induce apoptosis in PC3 and DU145 cells	inhibit IκBα phosphorylation and degradation and reduce phosphorylated p65 protein levels in nuclear fractions	[93]
IκBα	Acacetin	decrease cell viability of DUI45; suppress tumor growth	inhibit the phosphorylation of IκBα to suppress Akt and NF-κB signaling pathways	[94]
p65 as well as tumor-associated inflammatory infiltration	Metformin	decrease cell proliferation of PC3; suppress tumor growth; repress PCa progression	suppress N-cadherin and p65 accumulation; inhibit the COX2/PGE2 axis	[95,96]
NF-κB DNA-binding activity	3,3′-diindolylmethane (DIM) (synergize Taxotere)	inhibit cell growth and induce apoptosis in C4-2B, and inhibit C4-2B bone tumor growth	decrease in survivin expression and NF-κB DNA-binding activity	[97]
IκBα and NF-κB DNA-binding activity	Betulinic acid	inhibit cell growth and induce apoptosis in PC3 cells	decrease IKK activity and phosphorylation of IκBα; inhibit DNA binding and nuclear levels of the NF-κB/p65	[98]
feedback loop between pro-inflammatory cytokines CXCL1/2 and NF-κB	Curcumin	inhibit cell growth and metastasis in a PC3 cell model	abolish the feedback loop between pro-inflammatory cytokines CXCL1/2 and NF-κB through inhibiting IKKβ activation and NF-κB nuclear translocation	[39]
CXCL2-CXCR2 pathway in TAMs	AZD5069	lead to PCa cell senescence and inhibition of tumor progression	disrupt the CXCL2-CXCR2 pathway and trigger re-education of TAMs toward a pro-inflammatory state	[53]
IL8-stimulated NF-κB signaling	HuMaxIL8 (Anti-IL-8 antibodies)	inhibit PCa cell growth	attenuate activation of NF-κB (p65)	[37,99]
lncRNA-PCAT1-PHLPP/FKBP51/IKKα complex	lncRNA-PCAT1 shRNA	inhibit androgen-independentcell growth and CRPC progression	Prevent activation of AKT as well as NF-κB signaling	[35]
subunits of the IKK complex	lncRNA-DRAIC	inhibit cell invasion, soft agar colony formation, and tumor growth	inhibit interaction with the IKK complex, the phosphorylation of IκBa, and the activation of NF-κB	[34]
IKKβ-mediated MMP-9 and urokinase-type plasminogen activator	Aspirin	suppress invasion and attachment of DU145 and PC3 cells	decrease in inhibitors of κB IκBα phosphorylation, NF-κB p65 to nuclear translocation and IKKβ activation, leading to reduced MMP-9 activity and uPA and PAI-1 expression	[81]
IL-7/Akt/NF-κB signaling	Thymoquinone	inhibit IL-7-induced tumor progression and metastatic invasion in DU145 cells	down-regulate p-Akt and NF-κB, reduce the levels of MMP-3 and MMP-7	[82]
MMP-2, MMP-9, and NF-κB activity	Diosgenin	inhibit proliferation, cell migration, and invasion in PC3 cells	reduce the activities and expression of matrix metalloproteinase-2 (MMP-2) and MMP-9; suppress JNK, ERK, and PI3K/Akt signaling as well as NF-κB activity	[100]

## Data Availability

Not applicable.
